# Endothelin‐1 and ET receptors impair left ventricular function by mediated coronary arteries dysfunction in chronic intermittent hypoxia rats

**DOI:** 10.14814/phy2.13050

**Published:** 2017-01-05

**Authors:** Jin‐Wei Wang, Ai‐Ying Li, Qiu‐Hong Guo, Ya‐jing Guo, James W. Weiss, En‐Sheng Ji

**Affiliations:** ^1^ Department of PhysiologyHebei University of Chinese MedicineShijiazhuangHebeiChina; ^2^ Department of BiochemistryHebei University of Chinese MedicineShijiazhuangHebeiChina; ^3^ Department of PharmacologyHebei University of Chinese MedicineShijiazhuangHebeiChina; ^4^ Division of PulmonaryCritical Care and Sleep MedicineBeth Israel Deaconess Medical CenterBostonMassachusetts

**Keywords:** Cardiac function, chronic intermittent hypoxia, coronary resistance, endothelial receptors, endothelin‐1

## Abstract

Obstructive sleep apnea (OSA) results in cardiac dysfunction and vascular endothelium injury. Chronic intermittent hypoxia (CIH), the main characteristic of OSAS, is considered to be mainly responsible for cardiovascular system impairment. This study is aimed to evaluate the role of endothelin‐1(ET‐1) system in coronary injury and cardiac dysfunction in CIH rats. In our study, Sprague–Dawley rats were exposed to CIH (FiO_2_ 9% for 1.5 min, repeated every 3 min for 8 h/d, 7 days/week for 3 weeks). After 3 weeks, the left ventricular developed pressure (LVDP) and coronary resistance (CR) were measured with the langendorff mode in isolated hearts. Meanwhile, expressions of ET‐1 and ET receptors were detected by immunohistochemical and western blot, histological changes were also observed to determine effects of CIH on coronary endothelial cells. Results suggested that decreased LVDP level combined with augmented coronary resistance was exist in CIH rats. CIH could induce endothelial injury and endothelium‐dependent vasodilatation dysfunction in the coronary arteries. Furthermore, ET‐1 and ET_A_ receptor expressions in coronary vessels were increased after CIH exposure, whereas ET_B_ receptors expression was decreased. Coronary contractile response to ET‐1 in both normoxia and CIH rats was inhibited by ET_A_ receptor antagonist BQ123. However, ET_B_ receptor antagonist BQ788 enhanced ET‐1‐induced contractile in normoxia group, but had no significant effects on CIH group. These results indicate that CIH‐induced cardiac dysfunction may be associated with coronary injury. ET‐1 plays an important role in coronary pathogenesis of CIH through ET_A_ receptor by mediating a potent vasoconstrictor response. Moreover, decreased ET_B_ receptor expression that leads to endothelium‐dependent vasodilatation decline, might be also participated in coronary and cardiac dysfunction.

## Introduction

Obstructive sleep apnea (OSA) is a high risk factor for cardiovascular diseases, such as hypertension, diastolic ventricular dysfunction,coronary heart disease and stroke(Gami et al. 2013; Maeder et al. 2016; Ozkececi et al. 2016). Chronic intermittent hypoxia (CIH), directly caused by recurrent episodes of pharyngeal collapse occurring during sleep, is one of the most important risks of cardiovascular diseases in OSA patients (Schillaci et al. 2015).

Evidence indicated that CIH could induce left ventricular dysfunction in OSA. Patients (Yang et al. 2011). The cardiac dysfunction and cardiomyocyte injury were also observed in several animal modes exposed to CIH (Chen et al. 2005, 2008). CIH‐induced cardiac dysfunction was related to hypertension, ventricular remodeling and cardiomyocyte injury (Buchner et al. 2011; Baguet et al. 2012). Research also indicated that impaired myocardial perfusion was combined with decreased coronary flow reserve and systemic abnormalities in endothelial function in OSA patients (Butt et al. 2011). Therefore, CIH‐induced cardiac dysfunction might be associated with a decreased coronary tone, and an intact coronary endothelium was proved to be important in regulating coronary blood flow during OSA (Hamilton et al. 2008). Evidences showed that the incidence of coronary artery disease is increased in OSA patients (Peker et al. 2006). Previous studies suggested that CIH was contributed to microvascular endothelial dysfunction and augmented vasoconstriction (Allahdadi et al. 2005; Buchner et al. 2011), which was associated with endothelial system. Also, coronary artery endothelial cells dysfunction after intermittent hypoxia was considered to be related to the increased A20 (tumor necrosis factor alpha‐induced protein 3), VCAM‐1 (vascular cell adhesion molecule 1) and HIF‐1*α*(hypoxia‐inducible factor 1 alpha) expression(Kaczmarek et al. 2013).

Endothelin‐1 (ET‐1), one of the biologically active substances produced by endothelium, plays an important role in the cardiovascular alterations induced by CIH. Patients with OSA have a higher plasma level of ET‐1 than healthy people (Gjorup et al. 2007). In CIH rats, circulating ET‐1 levels and the sensitivity of the vasoconstrictor response to ET‐1 were also increased (Kanagy et al. 2001; Brunner et al. 2006). Moreover, previous studies have shown that ET‐1 and ET receptors play a key role in CIH‐induced endothelial dysfunction and augmented vasoconstriction of aortas and pulmonary arteries in rats (Guo et al. 2013; Wang et al. 2013). Thus, ET‐1 may also contribute to the coronary dysfunction induced by CIH.

As a potent endogenous vasoconstrictor, ET‐1 acts via two receptors, ET_A_ and ET_B_ (Hans et al. 2008). In healthy humans, ET_A_ receptor activation by ET‐1 potently stimulates vasoconstriction (MacCarthy et al. 2001) which contribute to basal coronary vascular tone (Pernow et al. 1996). Moreover, it was reported that ET_A_ receptor mRNA content was greater in coronary arteries from eucapnic intermittent hypoxia rats than that in sham‐exposed rats (Allahdadi et al. 2008). Although ET_B_ receptors expressed in smooth muscle cells is contributed to vasoconstriction (Pernow et al. 1996), their primary contribution to vascular tone in the coronary circulation is not well characterized. In contrast, ET_B_ receptors in endothelial cells have beneficial effects on the vascular wall by clearing ET‐1 from plasma and stimulating the vasodilator nitric oxide (NO) generation (Shetty et al. 1993; Hynynen and Khalil 2006). Therefore, the elevated ET‐1 and ET receptors induced by CIH can lead to the negative cardiovascular sequelae of OSA patients (Wang et al. 2013).

## Materials and methods

### Animals

Sprague‐Dawley rats weighing 240–280 g (*purchased from Hebei Medical University, Shijiazhuang, China)* were used in this study. Animals were kept under a 12:12‐h light‐dark cycle and were allowed free access to standard chow and tap water. The investigation conformed to the Guide for the Care and Use of Laboratory Animals published by the US National Institutes of Health (NIH Publication No. 85‐23, revised 1996), and was approved by the animal Ethics and Use Committee of Hebei Science and Technical Bureau in the People's Republic of China.

### Drugs

ET‐1 and BQ123 were obtained from Alexis Biochemicals (San Diego, CA), and BQ‐788 from Tocris Bioscience (Bristol, UK). Acetylcholine, SNP and _L_ –NAME were purchased from Sigma (St Louis, MO). ET‐1 and BQ123 were dissolved in normal saline and stored at 220 uC. BQ788 was dissolved in dimethylsulfoxide (DMSO), with the final concentration of DMSO less than 0.01%. Preliminary experiments showed that, 0.01% DMSO did not affect coronary reactivity and left ventricular function in response to ET‐1.

### Chronic intermittent hypoxia exposure

Rats were separated randomly into two groups: the first group was exposed to CIH. During hypoxic exposure, animals were placed daily in commercial hypoxic chambers that were flushed with 100% N_2_ to inspired O_2_ fraction (FIO_2_) nadir of 9% for 1.5 min. The FIO_2_ gradually returned to 21% over the remainder of each cycle. In the second group, rats underwent identical handling and exposure, but chambers flushed with room air rather than 100% N_2_. The exposure cycle was repeated every 3 min for 8 h/day, 7 days/week for 3 week during the animal's sleeping hours. After the exposure cycle was completed, animals were randomly assigned to either physiological investigation or molecular studies.

### Isolated, perfused rat heart preparation

Rats were killed with an overdose of pentobarbital sodium(150 mg/kg, i.p.) and hearts were rapidly removed into ice‐cold perfusion buffer, cannulated via the aorta and then perfused in the Langendorff mode with Krebs–Henseleit (KH) solution gassed with 95% O_2_ and 5% CO_2_ (pH 7.4) at constant perfusion pressure of 80 mmHg at 37°C. KH buffer contained (in nmol/L): NaCl 118.3, NaHCO_3_ 25, KCl 4.7, KH_2_PO_4_ 1.2, MgSO_4_·7H_2_O 1.2, glucose 11.1and CaCl_2_ 1.8. A latex balloon connected to a pressure probe was inserted into the left ventricle and filled until the diastolic pressure reached a value of 5–10 mmHg. Left ventricular developed pressure (LVDP) was calculated as the difference between left ventricular systolic pressure (LVSP) and left ventricular end diastolic pressure (LVEDP), and maximal rates of pressure development and fall, +dp/dtmax and –dp/dtmax, as indexes of contraction and relaxation, heart rate (HR) and coronary flow were used to assess heart function by a recording‐and‐analysis system (Powerlab system, Australia) that fed into the computer. All hearts were allowed an equilibration period of at least 25 min before any additional treatments given.

After this period, the heart was perfused at constant flow conditions. The flow rate was adjusted in order to obtain the same coronary flow as in the preparation at constant pressure. The index of cardiac function was recorded as already described.

### Coronary resistance

To assess coronary resistance (CR), after 25‐min equilibration period at constant pressure, hearts from the normoxia and CIH groups were injected with either acetylcholine (ACh; 60 pmol) to measure the degree of endothelium‐dependent vasodilation or sodium nitroprusside (SNP; 600 pmol) to evaluate the degree of endothelium‐independent vasodilation. Doses of ACh and SNP were determined according to previous research (Mourmoura et al. 2014). The coronary resistance was determined by the relationship between coronary flow and perfusion pressure (mm Hg/mL/min)(Figueroa‐Valverde et al. 2012).

### Effects of ET_A_ and ET_B_ receptor antagonists and NO synthase inhibitor on ET‐1‐induced changes of cardiac function and coronary response

In constant pressure condition, after stabilization, ET‐1 (20 pmol) was injected consecutively from the aorta with a microinjection pump (RWD202, RWD Life Science Co.). After the cardiac function recovery, the ET_A_ receptor inhibitor (BQ123; 1 nmol), ET_B_ receptor inhibitor (BQ788; 1 nmol) and the NO synthase (NOS) inhibitor N^G^‐nitro‐L‐arginine methyl ester (_L_ ‐NAME; 1 nmol) were injected into the aorta. About 15 min later, when the inhibitors worked completely, another 20 pmol ET‐1 was perfused, then the changes of cardiac function were recorded.

In another part of the experiments, effects of coronary flow on cardiac function were evaluated at constant flow. The flow rate was adjusted to the same as in the constant pressure conditions. After 25 min stabilization, ET‐1 (20 pmol) was injected and cardiac index was recorded. The doses of ET‐1 and inhibitions were selected on the basis of preliminary experiments in order to make the cardiac function changes achieve the maximum degree without impairment.

### Hematoxylin and eosin(H&E) staining and Immunohistochemistry

In order to assess the extent of coronary arteries injury after CIH, the ventricular tissue was fixed in 4% formaldehyde and paraffin embedded. For histological analysis, 6 *μ*m thick sections were stained with hematoxylin and eosin. The coronary arteries from the heart tissue were then observed under an optical microscope (Olympus BX41, Tokyo, Japan) at a magnification of 400 ×  for histological changes. In order to address the cellular localization of ET‐1, ET_A_ and ET_B_ receptors in coronary arteries,heart tissue was fixed as described. 5 *μ*m thick sections were immunohistochemically stained against ET‐1(EDN1 polyclonal antibody; Bioworld, USA; dilution 1:100), ET_A_ (ETAR polyclonal antibody; Bioworld, USA; dilution 1:100), ET_B_ (EDNRB Antibody; Affinity, USA; dilution 1:100). The expression and localization of ET‐1, ET_A_ and ET_B_ were determined by microscopic observation of the brown peroxidase in coronary artery at a 400 ×  magnification.

### Detection of ET‐1, ET_A_, ET_B_ and eNOS expression using western blot

The isolated hearts from normoxia and CIH rats were immersed in cold buffer solution, and then the coronary arteries were quickly freed from the heart under a dissection microscope and frozen in liquid nitrogen. Tissues were homogenized and lysed with RIPA lysis buffer containing 100 mg/mL PMSF and 1 mg**/**mL aprotinin. lysate was centrifuged at 12,000 rpm for 20 min at 4°C and the supernatant protein content was measured to detect ET‐1, ET_A_, ET_B_, eNOS and GADPH. Equal amounts of protein were denatured and subjected to electrophoresis on a sodium dodecylsulfate–8% polyacrylamide gel and transferred to nitrocellulose membrane (Millipore, USA). The membrane was then incubated with primary antibodies against ET‐1 (1:500; Genetex, Irving, CA), ET_A_ (1:1000; Enzo, New York, NY), ET_B_ (1:1000; Enzo) or eNOS (1:1000; Becton Dickinson, Franklin Lakes, NJ) overnight at 4°C. Equal protein loading was confirmed by blotting membranes with an antibody against GAPDH (glyceraldehyde 3‐phosphate dehydrogenase). The bands were visualised by an enhanced chemiluminescence (ECL) system (Fuji, Japan).

### Measurement of NO in coronary artery

The NO concentration in coronary arteries was determined by measuring the total nitrate and nitrite concentrations (Jian Cheng Biological Engineering Institute, Nanjing, China). This assay determined total NO based on the enzymatic conversion of nitrate to nitrite by nitrate reductase. The reaction was followed by the colorimetric detection of nitrite as an azo dye product of the Griess reaction. The absorbance of the mixture at 550 nm was determined by a microplate reader.

### Statistical Analysis

Results were presented as means ± SEM. Differences between normoxia and CIH groups were statistically analyzed with independent‐Samples T‐Test. All statistical analyses were performed using SPSS 13.0 (SPSS Inc., Chicago, IL). Two‐sided *P *<* *0.05 was considered significant.

## Results

### Effects of CIH on cardiac function of perfusing heart

The Table 1 depicts cardiac function of the isolated heart in baseline. The left ventricular developed pressure(LVDP), coronary flow (CF), +dp/dt_max_ and −dp/dt_max_ were decreased in the CIH group (7.5%, 26.2%, 17.8% and 31.5%, compared to the normoxia group, respectively, *P *=* *0.024, 0.042 and *P *<* *0.01, respectively), suggesting an damaged contractile function. Conversely, the coronary resistance (CR) was significantly increased in CIH rats (26.5% compared to the normoxia group, *P *=* *0.033). There were no differences in HR and LVEDP between the two groups.

**Table 1:** Baseline data for LVDP, LVEDP, heart rate, +dP/dt_max_, ‐dp/dt_max_ and coronary flow in isolated hearts from normoxia and CIH group

	LVDP	LVEDP	+dP/dt_max_	−dP/dt_max_	HR	CF	CR
	mmHg	mmHg	mmHg/sec	mmHg/sec	beats/min	mL/min	mmHg/mL/min
Normoxia	139.2 ± 3.14	8.7 ± 2.3	4558.3 ± 104.5	−3190 ± 64.1	280 ± 25	16.4 ± 4.2	5.2 ± 0.5
CIH	128.8 ± 3.03*	9.2 ± 1.7	3373.7 ± 85.9**	−2186 ± 53.6**	271 ± 29	12.1 ± 1.3*	7.0 ± 0.6*

Values are means ± SEM; *n* = 8.

CF, coronary flow; CIH, Chronic intermittent hypoxia; CR, coronary resistance; HR, heart rate; LVDP, left ventricular developed pressure; LVEDP, left ventricular end diastolic pressure.

*P <* *0.05 compared to normoxia group; **P <* *0.01 compared to normoxia group (independent‐Samples T Test).

### Effects of CIH on coronary resistance

**Figure 1:**
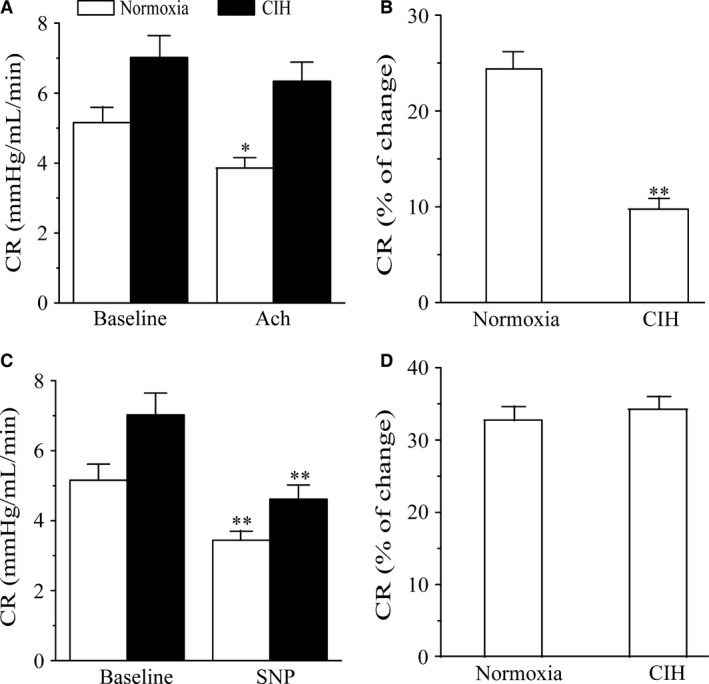
Effects of ACh and SNP on Coronary resistance (CR) of isolated hearts from normoxia and CIH groups. (A) CR in normoxia and CIH group before and after the injection of ACh. *P < 0.05, compared with baseline. (B) percent change in CR induced by ACh. **P < 0.01, compared with normoxia group. (C) CR in Normoxia and CIH group before and after the injection of SNP. **P < 0.01, compared with baseline. (D) percent change in CR induced by SNP. All data were means ± SEM. *n* = 8.

To determine whether CIH affects endothelium‐dependent or independent vasodilation in rat coronary artery, the responses to ACh and SNP in coronary arteries from normoxic and CIH rats were shown in Figure 1. Coronary resistance was significantly higher in animals exposed to CIH than that in normoxia rats (*P *=* *0.033). After treated with endothelium‐dependent vasodilator ACh, the coronary resistance in normoxia group was significantly decreased (*P *=* *0.037), but there were no significant change in CIH group (*P *=* *0.429; Fig. 1A). Moreover, the percentage of CR change in CIH group was higher compared with normoxia rats (*P *<* *0.01; Fig. 1B). Also, the endothelium‐independent vasodilator SNP induced a marked decrease of coronary resistance in both groups of animals (*P *<* *0.01; Fig. 1C), but the percentage of CR change was not affected by CIH exposure (*P *=* *0.467; Fig. 1D).

### Effects of CIH on ET‐1‐mediated CR and LVDP changes

**Figure 2:**
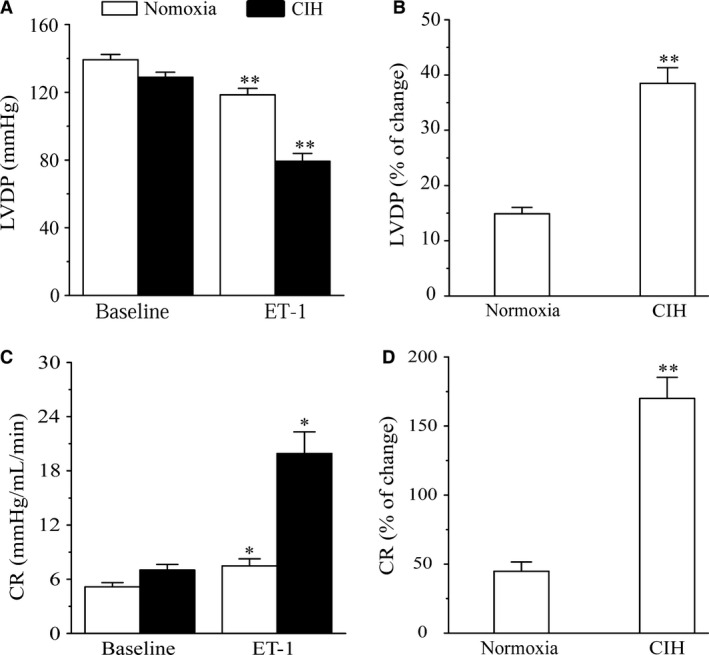
Effects of ET‐1 on LVDP and CR of Normoxia and CIH rats. (A) ET‐1 on LVDP in normoxia and CIH rats; **P < 0.01 compared to baseline (student's t‐test). (B) Change percentage of LVDP. **P < 0.01 compared to normoxia group. (C) ET‐1 on CR in Normoxia and CIH rats; *P < 0.05 compared to baseline. (D) Change percentage of CR. Values are expressed as means ± SEM. *n* = 8.

ET‐1 caused a significant decrease of LVDP (*P *<* *0.01) and an obvious increase of CR (Normoxia: *P *=* *0.028; CIH: *P *=* *0.011) in both groups (Fig. 2A, C), and the change percentage of LVDP and CR. was markedly higher in CIH rats than that in normoxia rats (*P *<* *0.01; Fig. 2B, D).

### Effects of ET_A_ and ET_B_ receptor antagonists on ET‐1‐induced CR and LVDP changes

**Figure 3:**
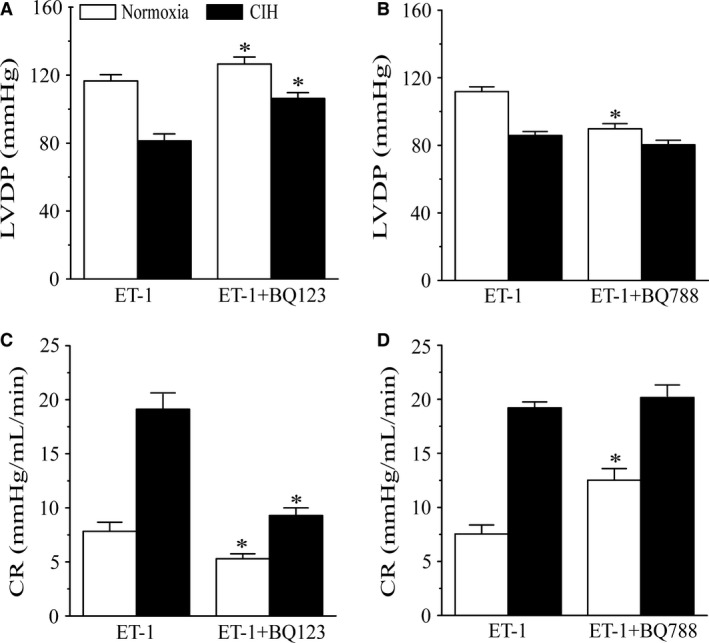
Effects of BQ123 and BQ788 on LVDP and CR in isolated hearts from normoxic and CIH rats. (A, C) 20 pmol ET‐1‐induced LVDP and CR changes were inhibited by 1 nmol BQ123. (B, D) 20 pmol ET‐1‐induced LVDP and CR changes were increased in normoxia rats by 1 nmol BQ788; All data are expressed as means ± SEM. *n* = 8.*P < 0.05 compared to the first ET‐1 induced minimum LVDP and maximal CR.

The LVDP and CR changes after ET‐1 administration were mostly inhibited by the ET_A_ receptor antagonist BQ123 in both groups (all *P < *0.05, Fig. 3A, C). However, the ET_B_ receptor antagonist BQ788 markedly augmented ET‐1‐mediated LVDP decrease and CR increase in normoxia rats (all *P *<* *0.05; Fig. 3B, D). The ET‐1‐induced decrease in LVDP (and increase in CR was not affected by BQ788 in CIH group (Fig. 3B, D).

### Effects of _L_‐NAME on ET‐1‐induced CR and LVDP changes

**Figure 4:**
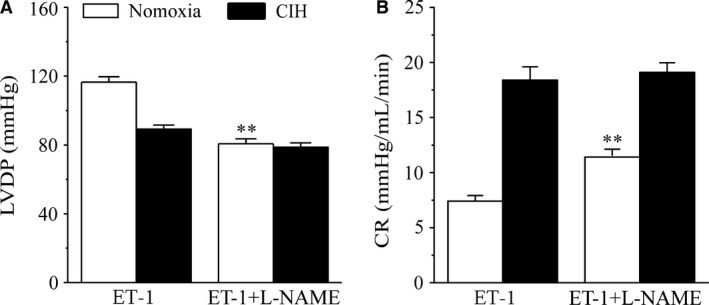
Effects of L‐NAME on LVDP and CR in isolated hearts from normoxic and CIH rats. A: Effects of 20 pmol ET‐1 on LVDP, with and without prior administration of 1 nmol L‐NAME. B: Effects of 20 pmol ET‐1 on CR, with and without prior administration of 1 nmol L‐NAME. All values are expressed as means ± SEM. *n* = 8. **P < 0.01 compared to the first ET‐1 induced LVDP and CR change.

The NO synthase (NOS) inhibitor _L_–NAME significantly enhanced ET‐1‐induced LVDP decrease and CR increase in normoxia group (*P *<* *0.01, Fig. 4A, B). However, in CIH group, there were no significant differences of LVDP (*P *=* *0.051) and CR (*P *=* *0.77) changes (Fig. 4A, B).

### Effects of ET‐1 on LVDP at constant coronary flow

**Figure 5:**
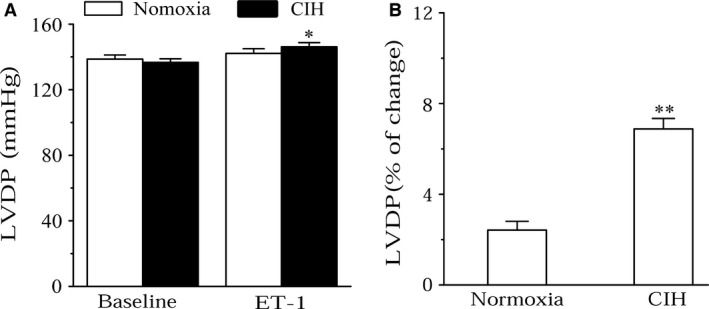
ET‐1‐mediated LVDP changes at constant coronary flow. (A) Effects of ET‐1 on LVDP in normoxic and CIH rats; *P < 0.05 compared to baseline in CIH group. (B) Change percentage of LVDP after injection of ET‐1; **P < 0.01 compared to normoxia group. All data were means ± SEM. *n* = 8.

To determine the role of myocardial contractility in 20 pmol ET‐1‐induced LVDP decrease, the coronary flow was controlled at 15 mL/min, nearly the same coronary flow as in the preparation at constant pressure. There was no significant difference in LVDP level between the two groups in baseline (Fig. 5A). And this experiment showed that 20 pmol ET‐1 could induce a little increase of LVDP in normoxia rats, but there was no significant difference compared with baseline (*P *=* *0.388; Fig. 5A). In the CIH group, 20 pmol ET‐1 markedly increased the LVDP from isolated hearts (*P *=* *0.017; Fig. 5A), and the percentage of LVDP change induced by ET‐1 in CIH rats was greater than that in normoxia group (*P *<* *0.01; Fig. 5B).

### Histological changes in coronary arteries

**Figure 6:**
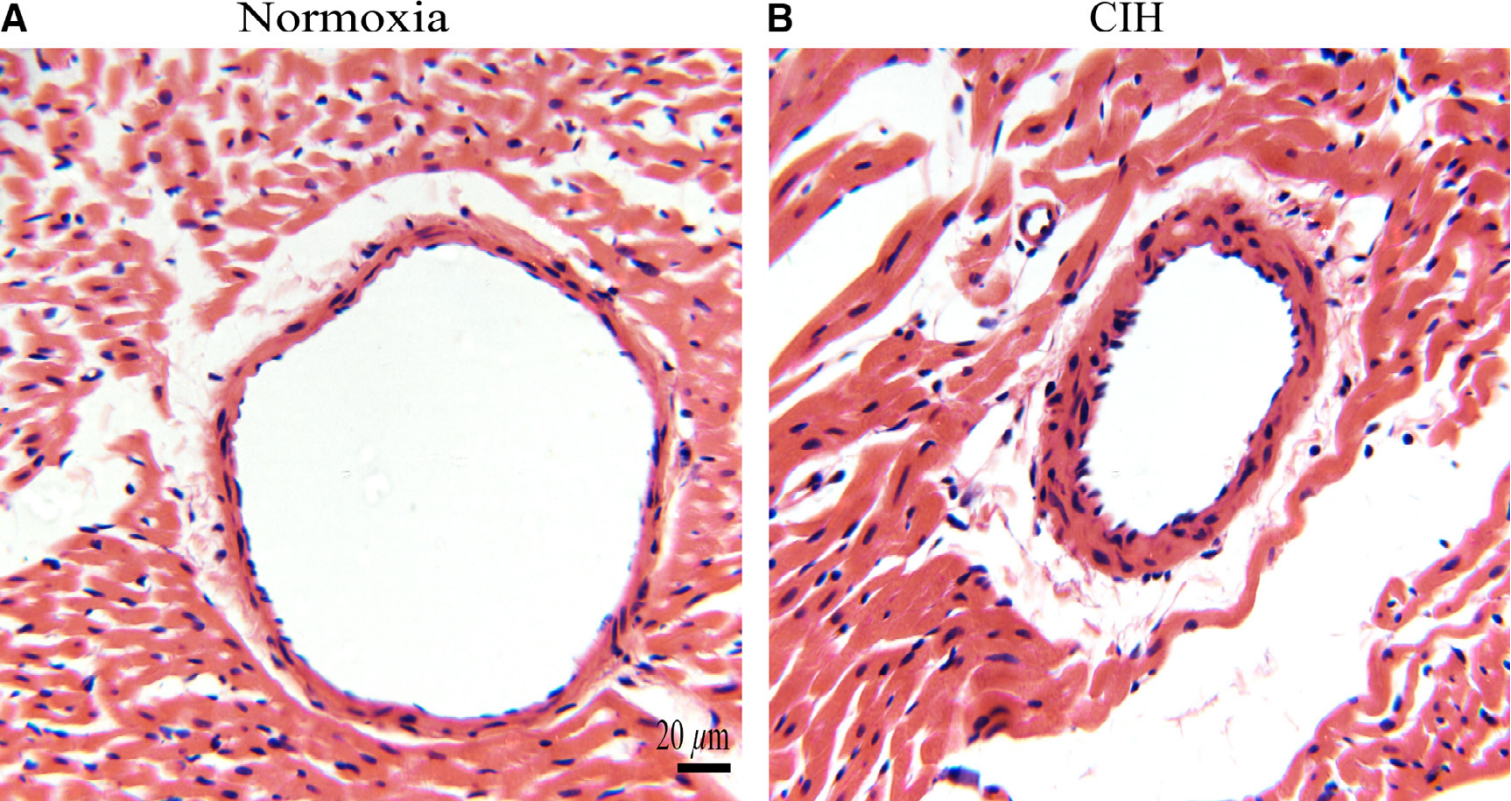
Effect of chronic interment hypoxia on the histopathology of coronary arteries. (A) Coronary histology of normoxia rats with intact endothelial cells. (B) Coronary histology of CIH rats with injury endothelial cells (magnification 400×).

In normoxia group, intima of coronary artery was smooth with the regularly shaped endothelia cells. And the wall thickness in coronary was normal with intact intima, media and adventitia. Coronary arteries in CIH rats exhibited obvious histopathological changes in endothelial layer with cellular enlargement and edema, and several endothelial cells tend to exfoliate. Moreover, the coronary arteries in CIH rats had an increased wall thickness with tissue edema in intima, media, and adventitia (Fig. 6).

### Coronary arteries expression of ET‐1, ET_A_, and ET_B_ in normoxia and CIH rats

**Figure 7:**
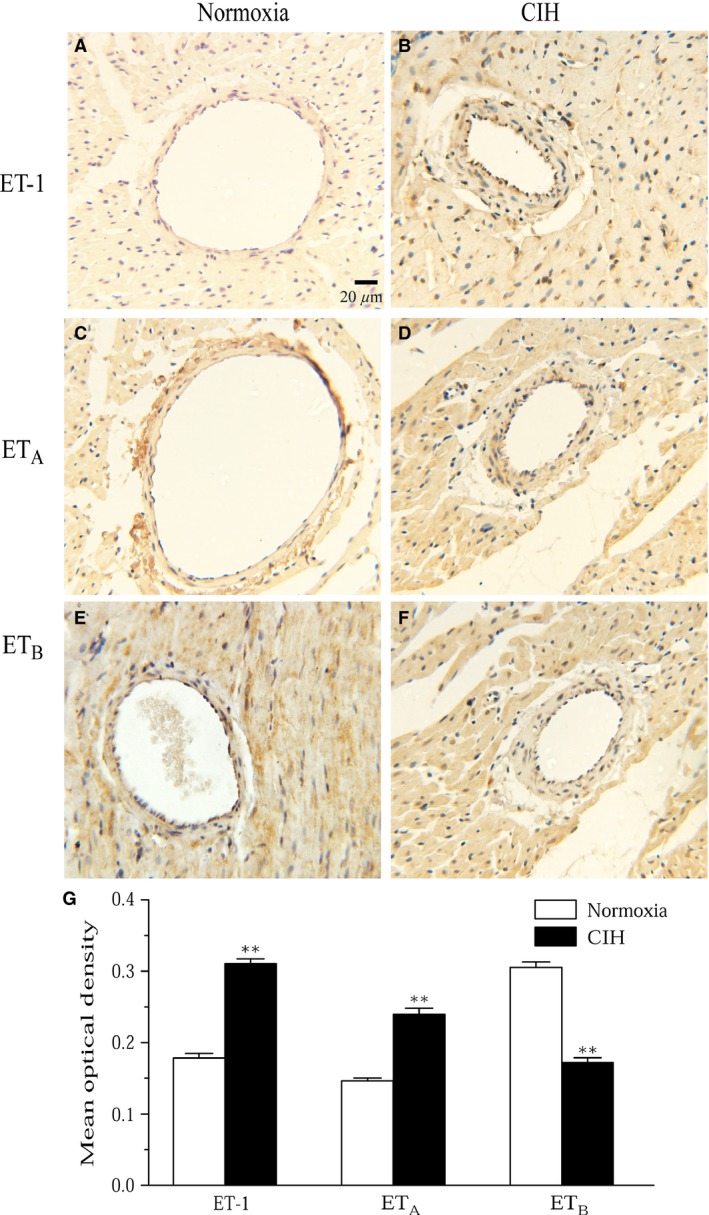
Coronary arteries expression and localization of ET‐1 and ET‐1 receptors in heart tissues from normoxia and CIH rats (magnification 400×). The dark brown color represents positive staining for ET‐1 and ET‐1 receptors. (A–F) Immunostaining of histological sections from normoxia (A, C, E) and CIH (B, D, F) rats for ET‐1, ET_A_ and ET_B_. (G) Average optical density of histological sections from normoxia and CIH rats for ET‐1, ET_A_ and ET_B_. Data were presented as Means ± SEM. *n* = 4. **P < 0.01 compared to normoxia group.

**Figure 8:**
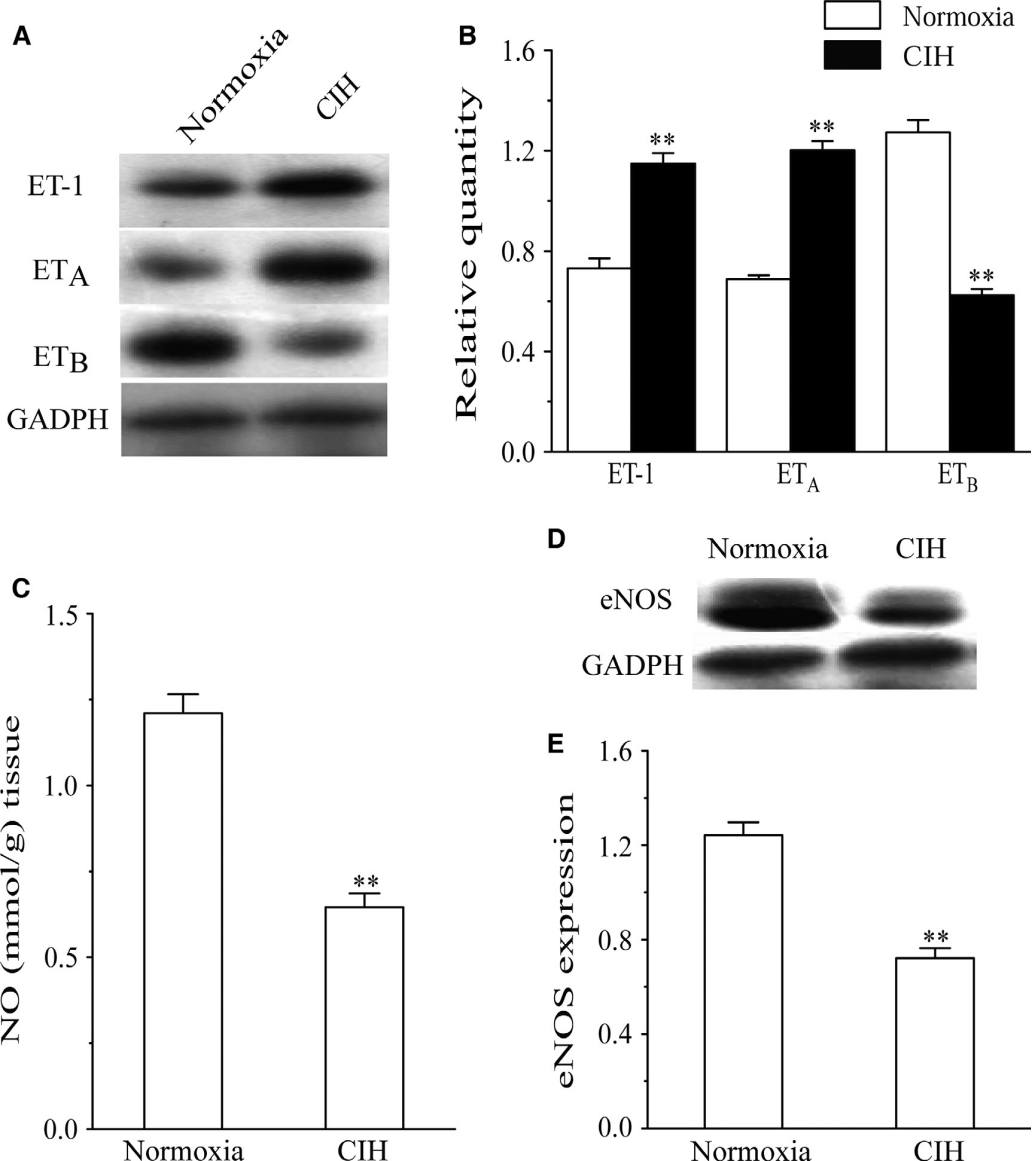
Effects of CIH on protein expression and NO production in coronary arteries from normoxia group and CIH group. (A, B) Western blot analysis of ET‐1 and ET receptors expression. (C) NO content in coronary arteries. (D, E) Western blot analysis eNOS protein expression. Results are expressed as means ± SEM of six subjects in each group. *n* = 8. **P < 0.01 compared with normoxia group.

Immunohistochemical staining was used to detect ET‐1 and ET‐1 receptors expression in rat coronary artery tissue (Fig. 7). Coronary arteries in CIH rats showed a strong positive staining pattern of ET‐1 on the endothelial layer and smooth muscle cell layer of the vessel walls compared with normoxia rats (Fig. 7A, B). Positive ET_A_ receptors staining were predominantly distributed in the smooth muscle cells of coronary arties from normoxia rats, which increased after CIH exposure (Fig. 7C, D). Coronary vessels from normoxia and CIH rats showed a similar positive staining pattern of ET_B_ on the smooth muscle cell layer. However, CIH‐induced vessels showed very light staining for ET_B_ located on endothelium compared with normoxia group (Fig. 7E, F). Moreover, Protein levels of ET‐1 and ET‐1 receptors were determined by western blot analysis. There was a significant upregulation of ET‐1 and ET_A_ receptors in CIH coronary arteries compared with normoxia group. However, coronary arteries from CIH rats showed substantially less density of ET_B_ protein bands as compared to normoxia rats (Fig. 8A, B). These data were analyzed with internal loading controls of GADPH.

### Effects of CIH on Rat coronary arteries NO production and eNOS Expression

To determine the effect of CIH on endothelia NO release in coronary arteries, the eNOS expression level and NO production in coronary vessels were measured. We observed a significant decrease in the eNOS expression levels in CIH group compared with normoxia group (Fig. 8D, E). Meanwhile, the NO levels of coronary arteries were reduced in CIH group (Fig. 8C).

## Discussion

The results of this study suggested that CIH‐induced cardiac dysfunction was associated with an increased coronary resistance mediated by ET‐1. The response to ET‐1 on coronary arteries was enhanced more in CIH rats compared to normoxia rats. Meanwhile, the augmented expression of ET_A_ receptor largely contributed to coronary dysfunction by causing a potent vasoconstrictor response in the coronary arteries of hearts from CIH rats. Furthermore, the damage of coronary artery endothelial cell, the decreased endothelial ET_B_ receptor expression and the reduction in NO release play an important role in enhancing coronary vasoconstriction that caused myocardial ischemia and hypoxia after CIH exposure, further leading to the failure of myocardial contractile function, cardiac enlargement and lower compliance (Song et al. 2014).

In this study, we estimated the isolated heart function from rats after exposed to CIH for 3 weeks. Results showed that the left ventricular function was decreased with a reduced coronary flow and an augmented coronary resistance. Thus, the hypoxia conditions in the experiment eventually impaired the cardiac function and coronary tone, which consist with the pathology observed in OSA patients (Butt et al. 2011). In addition, intermittent hypoxia was shown to reduce the endothelium dependent vasodilating capability of coronary arteries by ACh treatment. However, the endothelium‐independent vasodilation capability in coronary vessels was not affected by CIH exposure after SNP injection. These results indicated that the decreased Ach‐response of the CIH rats was due to an injury of endothelial cells. Moreover, histology changes showed obvious injury of endothelia cell in coronary arteries from CIH rats. As is known, the major endothelium‐derived vasoactive substances are ET‐1 and NO, which regulate the physical and biochemical properties of the coronary arteries and affect vascular contractility (Martinez et al. 2003). Thus, it is necessary to determine the role of ET‐1 and NO in coronary dysfunction induced by CIH.

In our study, 20 pmol ET‐1 induced a significant coronary vasoconstriction combined with a poor LVDP level in isolated hearts from normoxia and CIH rats at constant pressure condition, which was in contrast with the fact that ET‐1 could enhance myocardial contractility (Drawnel et al. 2012). We presumed that decreased LVDP in constant pressure was mainly induced by the augmented constriction of coronary arteries and the reduced coronary flow. The hypothesis was confirmed that when we forced the coronary flow in a constant level, the LVDP level was not affected by 20 pmol ET‐1 in normoxia group. However, 20 pmol ET‐1 induced augmented heart contractility in CIH rats, which might be due to the upregulation of ET_A_ receptors in myocardium induced by CIH. Thus, the ET‐1 mediated LVDP decrease was due to the augmented coronary vasoconstriction, and it would be interesting to investigate the mechanism of ET‐1‐mediated coronary injury in CIH rats.

Our study found that the ET‐1‐induced vasoconstrictor response and LVDP changes were almost completely inhibited by the ET_A_ receptor antagonist BQ123, which indicated the prominent role of ET_A_ receptor in ET‐1‐induced coronary constriction. However, the increased coronary resistance mediated by ET‐1 in CIH group was greater than normoxia group, suggesting that ET_A_ receptor expression was unregulated by CIH exposure. Furthermore, immunohistochemical and western blot results has demonstrated that ET‐1 and ET_A_ receptor expression in coronary arteries from CIH rats were exaggeratedly increased compared with normoxia rats. Indeed, these results were consistent with previous studies of ET‐1‐mediated artery and pulmonary dysfunction after CIH exposure (Guo et al. 2013; Wang et al. 2013), but few studies has reported ET‐1 and ET receptors changes in coronary arteries after CIH.

Although ET_A_ receptor was demonstrated to be the major receptors in modulating ET‐1‐induced vasoconstriction (Cannan et al. 1995), ET_B_ receptor also played an important role in regulating coronary arteries tone. Endothelial ET_B_ receptors were considered to mediate vasorelaxation in coronary arteries under physiological conditions. In contrast, ET_B_ receptors located in coronary smooth muscle cells (SMC) were considered to cause vasoconstriction (Cherng et al. 2009; Skovsted et al. 2012). In this study, BQ788 markedly aggravated ET‐1‐induced coronary constriction and augmented LVDP decreases in isolated hearts from normoxia rats,suggesting that BQ788 effectively inhibited ET_B_ receptors on endothelial cells. However, there were no significant changes in CIH rats after BQ788 injection, indicating the injury of endothelial ET_B_ receptors in coronary arteries after CIH. This result was different from our previous research that BQ788 could significantly reduce ET‐1‐mediated contractile responses in aortas and pulmonary arteries (Guo et al. 2013; Wang et al. 2013). Indeed, as an ET_B_ receptor inhibitor, BQ788 inhibited ET_B_ receptors on both vascular endothelial and smooth muscle cells to suppress endothelium‐dependent dilation and smooth muscle‐dependent constriction, and the final level of tone was the sum of these two responses. In coronary arteries, smooth muscle ET_B_ receptors contributed little to the ET‐1 response (Cherng et al. 2009). Thus, endothelial ET_B_ receptors might play the major role in regulating coronary function. But in aorta and pulmonary arteries, smooth muscle ET_B_ receptor might play a more important role than that in coronary arteries. Decreased endothelial ET_B_ receptors expression in coronary arteries of CIH rats supported the results of our experiment, wherever expression of the ET_B_ receptors located in coronary smooth muscle cells were not significantly reduced compared to normoxia rats, and the total number of ET_B_ receptors expression was decreased represented by western blot analysis. These results suggested that the function of ET_B_ receptors located in endothelia in coronary arteries was impaired after CIH exposure. Although several studies reported that ET_B_ receptors in SMC of coronary arteries have been demonstrated increased in several diseases such as experimental congestive heart failure (Cannan et al. 1996) and in patients with ischemic heart disease (Wackenfors et al. 2004), this study did not find significant changes of ET_B_ receptors in SMC of coronary arteries induced by CIH.

It is widely accepted that NO plays a significant role in cardiovascular regulation through modulation of vascular tone and endothelial function (Ignarro 1989; Grossini et al. 2014). NO is a primary endothelium‐derived relaxing factor regulating vascular resistance. Interaction between ET‐1 and NO has been demonstrated occurring in coronary arteries. Moreover, NO inhibitor has been shown to result in an augmented vasoconstriction induced by ET‐1 in the coronary arteries of rats (Wang et al. 1994). Endothelial NO synthase (eNOS), the major NOS isoenzyme expressed in endothelial cells, constitutively catalyzes the formation of NO from the amino acid L‐arginine (Umans and Levi 1995). Activation of endothelial ET_B_ receptors results mostly in increased endothelial nitric oxide (NO) synthase (eNOS) activity and NO release (Katakam et al. 2006). The NOS inhibitor _L_‐NAME markedly augmented ET‐1‐induced coronary artery contraction in normoxia group, implying that NO could counteract the ET‐1‐induced vasoconstriction. However, _L_‐NAME has no significant effect on ET‐1‐induced vasoconstriction in CIH group, and the diminished NO production in coronary arteries was found in CIH rats. Thus, it appears that vasoconstriction by ET‐1 may contribute to the reduction of NO release induced by CIH. Furthermore, this study has shown that eNOS protein expression in coronary arteries was significantly decreased in CIH rats, indicating that the decreased NO release was at least partly due to the down‐regulated expression of eNOS, which may be associated to endothelial ET_B_ receptor change induced by CIH.

There are some defects in our study. (1) Endothelial injury in coronary arteries may induce atherosclerosis, and previous studies have reported that OSA patients were combined with coronary atherosclerosis (Weinreich et al. 2013), but our study did not test the indicators related to coronary atherosclerosis in the CIH model. (2) Our study only used the male rats to stimulate CIH model, the effects of CIH on coronary and cardiac function of female rats were not determined. (3) It would be important to clarify the signal pathway that mediated the coronary dysfunction induced by ET‐1 and ET receptors in OSA patients and CIH models. We need a further study to reveal it.

In conclusion, our findings demonstrate that CIH‐induced cardiac dysfunction is associated with coronary injury, especially the endothelia injury. Moreover, the increased ET‐1 level and the changed expression of ET_A_ and ET_B_ receptors play an important role in the augmented coronary constriction and the impaired endothelia cell. Further researches are necessary to determine the detailed mechanism of CIH‐induced coronary dysfunction.

## Conflict of Interest

No conflicts of interest, financial or otherwise, are declared by the author(s).
